# Microalgae-Based Fluorimetric Bioassays for Studying Interferences on Photosynthesis Induced by Environmentally Relevant Concentrations of the Herbicide Diuron

**DOI:** 10.3390/bios12020067

**Published:** 2022-01-25

**Authors:** Gerardo Grasso, Giulia Cocco, Daniela Zane, Chiara Frazzoli, Roberto Dragone

**Affiliations:** 1 Istituto per lo Studio dei Materiali Nanostrutturati Sede Sapienza, Consiglio Nazionale delle Ricerche, P. le Aldo Moro 5, 00185 Rome, Italy; daniela.zane@cnr.it (D.Z.); roberto.dragone@cnr.it (R.D.); 2 Dipartimento di Scienze e Tecnologie per l’Agricoltura, le Foreste, la Natura e l’Energia, Università degli Studi della Tuscia, 01100 Viterbo, Italy; dr.giulia.cocco@gmail.com; 3 Dipartimento Malattie Cardiovascolari e Endocrino-metaboliche, e Invecchiamento, Istituto Superiore di Sanità, Via Giano della Bella, 34, 00162 Rome, Italy; chiara.frazzoli@iss.it

**Keywords:** ecosystem health, ecotoxicology, herbicides, microalgae, chlorophyll fluorescence, Kautsky effect

## Abstract

The widespread agricultural use of the phenylurea herbicide Diuron (DCMU) requires the investigation of ecotoxicological risk in freshwater and soil ecosystems in light of potential effects on non-target primary producers and a heavier effect on higher trophic levels. We used microalgae-based fluorimetric bioassays for studying the interferences on the photosynthesis of a freshwater and soil model green microalga (*Chlamydomonas reinhardtii*) induced by environmentally relevant concentrations of the herbicide DCMU. Measurements of steady-state chlorophyll *a* (Chl-a) fluorescence emission spectra were performed; as well, the kinetics of the Chl-a fluorescence transient were recorded. Percentage indexes of interference on photosynthesis were calculated after comparison of steady-state and kinetic Chl-a fluorescence measurements of DCMU-exposed and control *C. reinhardtii* cell suspensions. The results obtained after 30 min exposure to the herbicide DCMU confirmed a significant inhibitory effect of DCMU 2 μg/L, and no significant differences between %ι values for DCMU 0.2 μg/L and 0.02 μg/L exposures. Positive %ε values from kinetic measurements of the Chl-a fluorescence transient confirmed the same interfering effect of 2 μg/L DCMU on PSII photochemistry in the exposed *C. reinhardtii* cell suspensions. Negative values of %ε observed for 0.2 and 0.02 μg/L DCMU exposures could be attributable to a presumptive ‘stimulatory-like’ effect in the photochemistry of photosynthesis. Short-term exposure to sub-μg/L DCMU concentration (≤0.2 μg/L) affects the photosynthetic process of the model microalga *C. reinhardtii*. Similar environmental exposures could affect natural communities of unicellular autotrophs, with hardly predictable cascading secondary effects on higher trophic levels.

## 1. Introduction

In recent years, the increasing use of phenylurea herbicides (PUHs) as well as residual activity PUHs and their degradation products in crops, soils, and freshwaters pose a serious risk to the environment and human health. Diuron (3-(3,4-dichlorophenyl)-1, 1-dimethylurea or DCMU) is one of the most relevant members of substituted phenylurea herbicides and a chemical of great agronomical importance worldwide [1]. DCMU is extensively used as a selective pre-emergence herbicide against mono- and dicotyledonous weeds in agricultural crop areas of citrus, grapes, cotton, sugar cane, alfalfa, and wheat. Furthermore, DCMU is used for general weed control in non-crop areas (e.g., roads, garden paths, railway lines, industrial sites, rights-of-way, around farm buildings, on irrigation, and drainage ditches) [2,3]. It can also be used as mildewcide, as an algaecide in commercial fish productions, and as antifouling in paint biocide production [4]. DCMU also showed its efficacy against mosses and as a soil-sterilizing agent [5].

Data on adsorption of PUHs by temperate soils are largely available, while very few data are available for tropical soils [6]. Studies about environmental fate and behavior highlighted that DCMU is relatively persistent in the environment; it is mobile in soil and relatively stable in water [3,7]. DCMU is prone to phenomena such as agricultural run-off, leaching (during rain events), and migration to both surface and groundwater aquatic environments. Available toxicity data suggest that DCMU can cause adverse effects on non-target organisms such as fish [8,9,10,11]. A knowledge gap still exists in the literature about DCMU toxicity on other non-target organisms such as amphibians [8].

Unicellular green algae are primary producers at the basis of the aquatic food chain, thus bearing a high ecological relevance. The ecological relevance of unicellular green algae implies that adverse effects on these microorganisms can negatively influence higher trophic levels, including zooplankton and fish [12]. As a consequence of increased DCMU (as for other photosystem II inhibitor herbicides) residues in the environment, acute adverse effects can occur on natural populations of unicellular autotrophs. To date, little is known about potential adverse ecological effects on a longer timescale. These effects can include chronic adverse effects on natural communities, cascading secondary effects on other communities’ ecological processes, and hardly predictable effects on different trophic levels and, ultimately, on ecosystems.

Recently, a model assessing the effect of high-frequency pulses (simulating exposure in agricultural applications and run-off) of DCMU exposure of freshwater benthic biofilms suggested that adverse impacts are very likely [13]. The influence of water flow conditions on DCMU bioaccumulation by freshwater biofilms has also been recently assessed [14]. The green microalga *Chlamydomonas reinhardtii* contributes to the primary production of a wide range of natural habitats, including freshwater (e.g., small pools, ditches, pelagic zone of lakes) and soils (e.g., temperate cultivated fields) [15]. In addition, *C. reinhardtii* has been widely used as a model organism for understanding the response to herbicides, the mechanisms behind herbicide action, as well as being a biologiacal indicator of pesticide toxicity in the environment [16]. Bioassays based on ecologically important and model organisms are crucial to study and screen chemical hazards in real-life matrices, as well as for diagnostic assessment of acute ecotoxicity.

In the present study, the freshwater and soil model microalga *Chlamydomonas reinhardtii* was used in microalgae-based fluorimetric bioassays to study acute interferences on photosynthesis following short-term (30 min) exposures to environmentally relevant concentrations of the herbicide DCMU (concentration range 0.02–2 μg/L). The concentration of 2 μg/L was close to 1.8 μg/L, i.e., the European maximum allowable concentration for inland surface waters (i.e., rivers and lakes and related artificial or heavily modified water bodies) as reported by Annex I, Part A: Environmental Quality Standards of DIRECTIVE 2013/39/EU [17]. The 0.2 μg/L concentration of DCMU corresponds to the annual average value for inland surface waters and other surface waters, and it is twice the maximum allowable concentration of a single pesticide in drinking water as regulated by the Drinking Water Directive (0.1 μg/L) [18]. With the purpose of investigating acute effects, two fluorescence-based bioassays were carried out: (i) steady-state chlorophyll *a* (Chl-a) fluorescence emission spectra; (ii) kinetics measurements of the chlorophyll *a* fluorescence transient. Chlorophyll *a* fluorescence is an information-rich tool that can provide interesting insights about the interfering effects of chemical stressors on photosystem II (PSII) photochemistry and the photosynthetic process in general [19].

## 2. Materials and Methods

### 2.1. Chlamydomonas reinhardtii Cell Suspensions

Axenic cultures of green microalga *Chlamydomonas reinhardtii* (CC-125 wild type 137c, mating type +, purchased from *Chlamydomonas* Resource Center, University of Minnesota, USA) were grown in TAP growth medium (Gibco^®^ TAP medium optimized for *Chlamydomonas* culture, purchased from Thermo Fisher Scientific, Waltham, MN, USA). *C. reinhardtii* liquid cultures (2:1 flask-to-medium ratio) was grown at 25 ± 1 °C in autoclaved sterile Erlenmeyer Flasks stoppered with autoclaved sterile cotton wool buds. Erlenmeyer Flasks were kept in an orbital shaker incubator with thermostatic cupola (mod. 711/CT +, ASAL Srl, Milan) under constant agitation (150 rpm) and continuous illumination provided by a 16 W cool white fluorescent tube placed 20 cm above the culture flasks (Figure S1).

Cell counting was carried out using a counting chamber and a Reichert-Jung MicroStar 110 microscope, Leica Microsystems Wetzlar, Germany. Measurements of optical density at a fixed wavelength of 750 nm (OD_750 nm_) were performed in quartz cuvettes with 1 cm optical path lengths against TAP medium as reference (UV2 UV/Visible, Unicam Instruments, Cambridge, UK). Seven serial dilutions were prepared using a one-week growing *C. reinhardtii* cell culture (range of cells/mL between 2 × 10^5^–1.2 × 10^6^ cells/mL). At the wavelength of 750 nm, the interferences due to light absorption by photosynthetic pigments are minimal [20]. The calibration curve was constructed by plotting OD_750 nm_ vs. 10^5^ cells/mL values (y = 0.0658 × −0.0311) (Figure S2). Good agreement (R^2^ = 0.9638) was found between OD_750 nm_ and *C. reinhardtii* cellular concentration.

### 2.2. Herbicide DCMU Solutions

A 2.5 g/L stock solution of DCMU (≥98% purchased from Sigma-Aldrich, Milano, Italy) was prepared in methanol (assay GLC ≥ 99.9%, obtained from Carlo Erba Srl, Italy), kept dark, and stored at 4 °C in a glass bottle. Before each photosynthesis-interference bioassay, dilutions with high-purity deionized water (Milli-Q system, Merck Millipore, Billerica, MA, USA) were carried out to obtain DCMU working solutions 10 times more concentrated than the final concentrations tested (i.e., 0.2, 2 and 20 μg/L).

Before each bioassay, DCMU stock solution was verified through spectrophotometric measurements at λ = 250 nm, which coincides with DCMU’s absorbance peak in water [21]. The spectrophotometric measurements (Unicam UV2 UV/Visible spectrophotometer, Unicam Instruments Ltd, Arbury Road, Cambridge, United Kingdom) were carried out against high-purity deionized water in quartz cuvettes with 1 cm optical path lengths at 25 °C on 1/200 dilution of DCMU stock solutions (λ = 250 nm; 0.798 ± 0.005 absorbance units).

### 2.3. Microalgae-Based Bioassays

#### 2.3.1. Measurements of In Vivo Steady-State Chl-a Fluorescence

**Figure 1:**
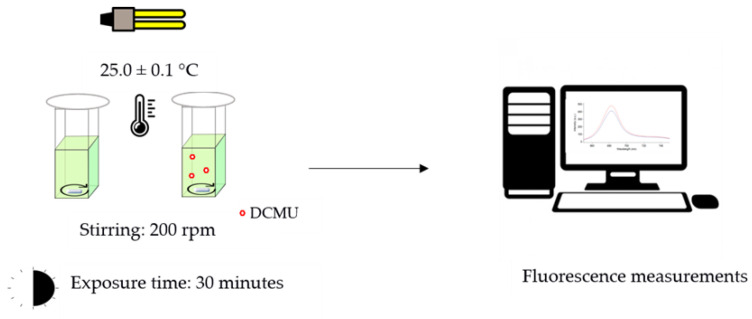
Schematic representation of experimental protocol used for the in vivo steady-state Chl-a fluorescence emission spectra.

Chlorophyll *a* fluorescent analysis has become a widely used technique to measure photosynthetic efficiency. Emission at 684 nm (F_684 nm_) is considered the main emission band associated with PSII at room temperature [22], mostly originating in PSII antenna complexes [23]. The increase in the F_684 nm_ value reflects changes in photosystem II photochemistry associated with an inhibition of photosynthetic activity [24]. Each bioassay was performed in triplicate following the same experimental set-up (Figure 1):

- Control microalgae cell suspensions: 2.70 mL of microalgae cell suspension (OD_750 nm_ of 0.20 ± 0.02) + 0.30 mL of high-purity deionized water.
- DCMU-exposed microalgae cell suspensions: 2.70 mL of microalgae cell suspension (OD_750 nm_ of 0.20 ± 0.02) + 0.30 mL of DCMU working solutions (10 times more concentrated than the final concentrations tested, i.e., 0.2, 2, and 20 μg/L).

All the microalgae cell suspensions were placed in quartz cuvettes with 1 cm optical path lengths at controlled temperature (25.0 ± 0.1 °C), under continuous light (16 W cool white fluorescent tube placed 20 cm above), and under constant magnet stirring (200 rpm). During the exposure time, the cuvettes were covered with 20 mm diameter cellulose filter discs (Whatman^®^ qualitative filter paper, Grade 1; Sigma Aldrich, Milan, Italy) to limit possible dust contamination of samples and water evaporation (<5%), while ensuring a proper gaseous exchange between air and microalgae cell suspensions. After 30 min, the fluorescence emission spectra were recorded at 25 °C using a Cary Eclipse spectrofluorometer (Varian, USA) both on DCMU-exposed microalgae cell suspensions and on blank microalgae cell suspensions (Figure S3). Spectrofluorometer was set as follows: excitation wavelength = 489 nm; range of emission wavelengths: 650 and 800 nm; excitation and emission slits 10 nm; scan rate 120 nm/min.

The comparison of F_684 nm_ values between exposed and control microalgae cell suspensions was used as an index to assess interference on photosystem II photochemistry, as previously described by Fai et al., 2007 [25], and calculated as follows:

% ι = [(F_684 nm exp_/F_684 nm blk_) − 1] × 100

where F_684 nm exp_ = mean of values of fluorescence emission at 684 nm (in arbitrary units) for DCMU-exposed microalgae cell suspensions, and F_684 nm blk_ = mean of values of fluorescence emission at 684 nm (in arbitrary units) for control microalgae cell suspensions.

#### 2.3.2. Kinetic Measurements of a Chl-a Fluorescence Transient

The Kautsky effect is a known biophysical phenomenon of Chl-a fluorescence transient induction (or OJIP transient) observed when dark-adapted photosynthetic samples are exposed to saturating light intensities. The Kautsky effect includes two phases: a first exponential (less than a second) and a second one slowly decaying (few minutes). The first phase is called ‘OJIP phase’, where ‘O’ stands for ‘origin’, i.e., the first measured minimal level, J and I are intermediate levels around 2 and 30 ms, respectively, and P is the peak. Changes in fluorescence intensity during the OJIP phase have been mainly associated with changes in redox states of components of the linear photosynthetic electron transport chain of photosystem II, as well as to the kinetics of redox reactions that occur through it [26].

Thus, the OJIP phase can be potentially used for the characterization of PSII photochemistry, the electron transport activity, as well as to monitor the inhibition effects of photosynthesis stressors, including the herbicide DCMU [27]. OJIP tests were performed using a fluorometer developed under the ‘Alert’ program [28] as part of the ‘Best’ prototype [29]. This six-sample chambers fluorometer uses a non-modulated (continuous) LED light source for excitation (λ_ex_ = 650 nm, intensity 127 μmol m^−2^ s^−1^, duration 11 s), λ_em_ = 690–715 nm. The spectra baseline was corrected with spectra of TAP growth medium.

**Figure 2:**
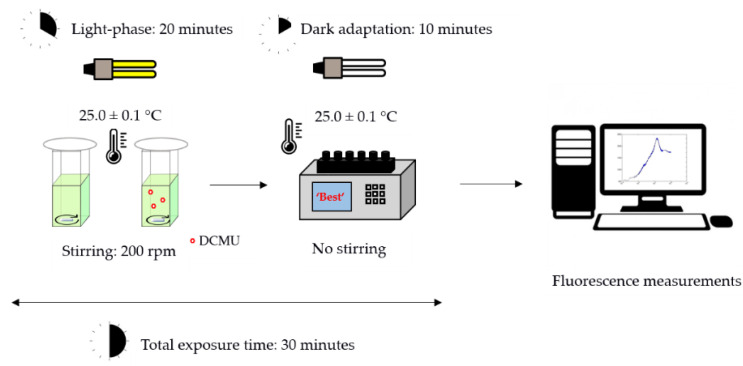
Schematic representation of experimental protocol used in the kinetic bioassays.

Each bioassay was performed in triplicate following the same experimental set-up (Figure 2). The microalgae cell suspensions were incubated for 20 min using same conditions described for in vivo steady-state fluorescence emission of Chl-a bioassays (Section 2.3.1, Figure 1). Then, microalgae cell suspensions were transferred into the six-sample chambers fluorometer and dark-adapted for 10 min before the fluorescence measurements. A scheme of the experimental protocol used in the kinetic measurements of Chl-a fluorescence transient is reported in Figure 2.

Analyses of Kautsky curves were performed using MatLab v7.8 (The MathWorks Inc., Natick, MA, USA). The following parameters of Kautsky curves were calculated:

- Fo (minimum of fluorescence intensity yield in the absence of photosynthetic light): calculated with data between 0.1 and 0.14 ms as the intercept of linear regression;
- F_M_ (maximum of fluorescence intensity);
- F_8 ms_ (fluorescence intensity at 8 ms): average fluorescence values acquired at 8.0 ± 0.1 ms;
- V_8 ms_ (the relative variable fluorescence at 8 ms) calculated as follows:
  V_8 ms_ = (F_8 ms_ − Fo)/(F_M_ − Fo)

The comparison of V_8 ms_ values between exposed and control microalgae cell suspensions was used as an index to assess interference on PSII photochemistry, and calculated as follows:

%ε = [(V_8 ms exp_ − V_8 ms blk_)/(1 − V_8 ms blk_)] × 100

where V_8 ms blk_ = mean of V_8 ms_ values for control microalgae cell suspensions and V_8 ms exp_ = mean of V_8 ms_ values for DCMU-exposed microalgae cell suspensions.

### 2.4. Data Analysis

Each bioassay was performed in triplicate on the same day for all the three DCMU concentrations to assess the repeatability of the measurements. Reproducibility was verified by performing bioassays on three different days, starting from freshly prepared DCMU working solutions and *C. reinhardtii* cell suspensions. Statistical differences between exposed and control *C. reinhardtii* cell suspensions were analyzed using two-way analysis of variance (ANOVA) without replication testing for randomized block design. Randomized block design of ANOVA testing helped to reduce possible confounding factors that may affect the comparison between data acquired in different days of testing, e.g., possible differences between working *C. reinhardtii* cell suspensions prepared on different days [30].

## 3. Results

### 3.1. In Vivo Steady-State Fluorescence Emission of Chl-a

**Figure 3:**
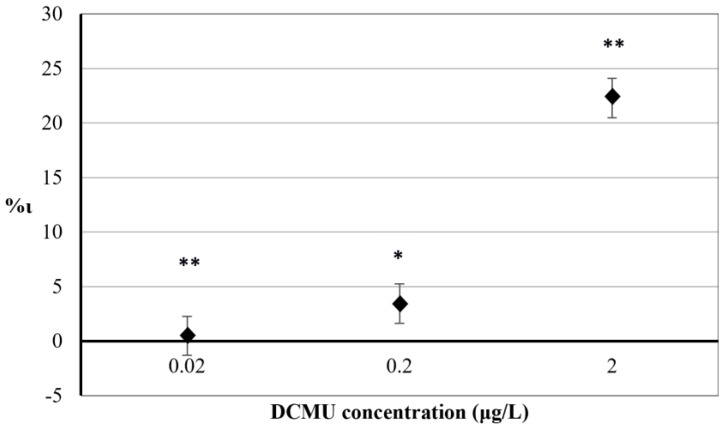
The comparison of F_684 nm_ values between exposed and control microalgae cell suspensions after 30 min exposure to DCMU. RSD ≤ 15%. Two–way ANOVA without replication testing was applied for statistical differences between means values of F_684 nm exp_ and F_684 nm blk_. Statistical significance of the results is indicated with asterisks (* *p* < 0.05; ** *p* < 0.01).

The results obtained for 30 min exposure to herbicide DCMU on the percentage increase in F_684 nm_ index (%ι) are reported in Figure 3.

A significant relationship between the mean variation of F_684 nm_ values in exposed and control *C. reinhardtii* cell suspensions was found at all concentrations tested (*p* < 0.05 for 2 μg/L exposure and *p* < 0.01 for 0.2 and 0.02 μg/L, respectively). Positive values of %ι indicate interference in PSII photochemistry in exposed *C. reinhardtii* cell suspensions (compared to control cell suspensions) due to the inhibitory activity of DCMU on the photosynthetic electron transport chain. Results from exposure of *C. reinhardtii* cells to 2 μg/L DCMU showed a significant inhibition (%ι = 22.28). Exposures to 0.2 μg/L and 0.02 μg/L DCMU showed no significant differences between %ι values (%ι = 3.48 and %ι = 0.48, respectively).

### 3.2. Chlorophyll a Fluorescence Transient

**Figure 4:**
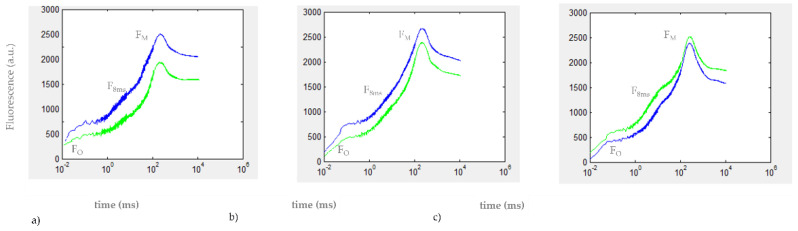
OJIP phase of the Kautsky curves recorded for (**a**) 0.02 μg/L DCMU, (**b**) 0.2 μg/L DCMU, and (**c**) 2 μg/L DCMU–exposed microalgae cell suspensions (green line). Blank microalgae cell suspensions are in the blue line. Fo (minimal fluorescence, first reliable fluorescence value); F_M_ (maximal fluorescence value at the peak of the OJIP curve under saturating illumination); F_8 ms_ (fluorescence value at 8 milliseconds). Baseline correction was performed using curves from TAP growth medium.

The Kautsky curves recorded after 30 min (20 min under light + 10 of dark adaptation) exposure to the herbicide DCMU are reported in Figure 4.

**Figure 5:**
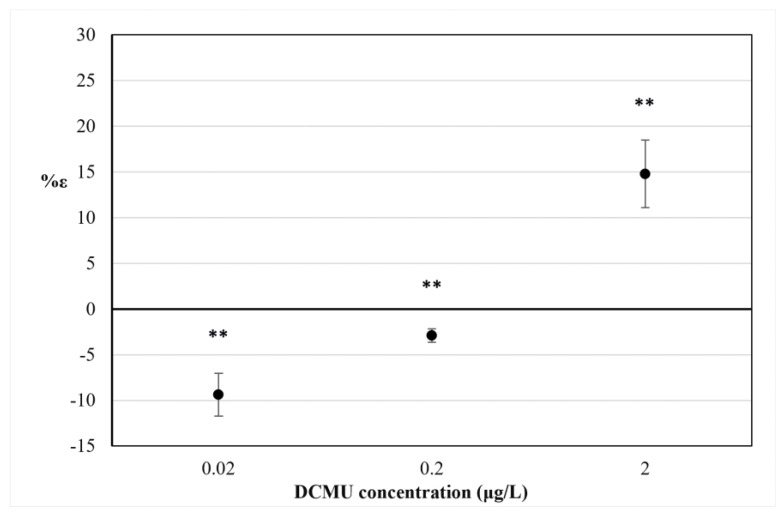
The percentage variation of V_8 ms_ index (%ε) after 30 min exposure to DCMU. RSD ≤ 15%. Two-way ANOVA without replication testing was applied for statistical differences between means values of V_8 ms exp_ and V_8 ms blk_. Statistical significance of the results is indicated with asterisks (** *p* < 0.01).

The percentage increase in V*_8 ms_* (%ε) is shown in Figure 5.

At all DCMU concentrations tested, a difference was found in the mean variation of V_8 ms_ values between exposed and control *C. reinhardtii* cell suspensions (*p* < 0.01). As for the results of in vivo steady-state fluorescence emission of Chl-a measurement (Section 3.2), a positive value of %ε for 2 μg/L DCMU exposure indicates interference in PSII photochemistry in exposed *C. reinhardtii* cell suspensions (compared to control cell suspensions), due to the inhibitory activity of DCMU on the photosynthetic electron transport chain. Negative values of %ε observed for 0.2 and 0.02 μg/L DCMU exposure correspond to a significant decrease in V_8 ms_ for exposed *C. reinhardtii* cell suspensions (compared to control cell suspensions) and could be attributable to a presumably ‘stimulatory-like’ interference on PSII photochemistry. Decreases in F_M_ values were observed for all *C. reinhardtii* cell suspensions exposed to sub-μg/L (≤0.2 μg/L) DCMU concentrations compared to control (Figure 4).

## 4. Discussion

Our results are consistent with the previous results obtained by Fai et al. (2007) for 3 μg/L DCMU exposures, using steady-state fluorescence emission spectra of Chl-a based bioassay [25]. Fai et al. (2007) worked with the green microalga *Selenastrum capricornutum* (also known as *Raphidocelis subcapitata* or *Pseudokirchneriella subcapitata*) that is used in the standard 72-hr growth inhibition assay for acute and chronic toxicity assessment [31]. Optimal response has been obtained after 30 min exposure of *S. capricornutum* cells to DCMU using a microplate-based protocol. The results of the percentage increase in F_684 nm_ (%ι) have shown a positive linear association (R^2^ = 0.8) with results from using the 72-h *S. capricornutum* growth inhibition assay [31]. Our method obtained results of a DCMU concentration 10-fold lower than those reported by Fai et al. (2007). With respect to the experimental protocol used by Fai et al. (2007) for *S. capricornutum*, it can be hypothesized that the exposure conditions of constant stirring and illumination adopted in our bioassays can promote the *C. reinhardtii* response to DCMU.

The fast kinetics of the Chl-a transient fluorescence and the polyphasic OJIP induction curve are thought to be largely determined by changes in the redox state of the primary PSII quinone (Q_A_), but also reflect interferences in the photosynthetic electron transport chain [32]. In particular, the parameters measured in our study F_8 ms_ and V_8 ms_ and its percentage increase (%ε) refer to the JI phase (approximately 3–30 ms) of the polyphasic OJIP induction curve, whose kinetics reflect the presence of (over)oxidized PQ molecules and the (partial) reduction in the PQ pool [33]. The positive value of the V_8 ms_ index observed for 2 μg/L DCMU exposures (%ε = 14.77%) in Figure 5 can be associated with the inhibition of PSII of *C. reinhardtii* cells. It is widely known that DCMU inhibits PSII, thus blocking the plastoquinone pool (PQ-pool) reduction through the impairment of the electron transfer between primary and secondary PSII quinones, Q_A_ and Q_B,_ respectively [34]. This impairment inhibits the so-called ‘state 1’ of the photosynthetic apparatus when the linear electron transfer (LET) from PSII to PSI occurs [35]. The LET is mostly active in the absence of DCMU, while in the presence of DCMU, the cyclic electron transport (CET) prevails over the LET, the so-called ‘state 2’ [36]. The decrease in fluorescence signals in *C. reinhardtii* cell cultures exposed to sub-μg/L DCMU concentrations (Figure 4) and negative %ε values observed for *C. reinhardtii* cell suspensions exposed to the same DCMU concentrations (Figure 5) could be associated with a presumptive early mechanism of acclimation to DCMU-induced stress at sub-μg/L doses. A decrease in excitation energy as fluorescence corresponds to a higher rate in energy utilization in photochemistry [37]. It could be hypothesized that partial inhibition of photosynthetic linear electron transport by DCMU at sub-μg/L concentrations (≤0.2 μg/L) can affect the modulating energy flux through the state transitions, inducing a ‘stimulatory-like’ mechanism of the photosynthetic process downstream of PSII. Although it is widely accepted that variations in the polyphasic OJIP induction curve are mostly due to changes in the redox state of the reaction center complex of PSII, fast kinetics of the Chl-a fluorescence transient are also affected by changes occurring in the overall photosynthetic apparatus [26]. The imbalance between LET and CET, induced by partial inhibition of photosynthetic linear electron transport induced by exposure to sub-μg/L DCMU concentrations (≤0.2 µg/L) and the parallel cyclic electron transport (CET) activity could influence PQ-pool redox poise and therefore induce the lowering fluorescence emission observed. The relationship between PQ-pool redox poise and LET/CET balance is widely accepted [32,36,38]. The PQ-pool redox poise can also influence state transitions [35] and modulate several redox-signaling pathways that can affect photosynthetic efficiency [39].

Therefore, the decrease in F_M_ values (Figure 4) and negative %ε values observed for *C. reinhardtii* cell suspensions exposed to sub-μg/L DCMU concentrations (Figure 5) could be attributed to the quenching of fluorescence due to an increased oxidation state of the plastoquinone (PQ) pool, as well as to a transition from LET to CET [40].

We can hypothesize the influence of these mechanisms on our results, but no data are available so far, and further measurements will be necessary to confirm this hypothesis. Moreover, other mechanisms could be involved. In *C. reinhardtii* wild type cells, the Calvin-Benson-Bassham cycle is partially active, although PSII was inhibited by DCMU, and it affects the number of electrons that can be allocated to CET [34]. The activity of recently identified stromal photosynthetic alternative electron transport pathways, such as flavodiiron proteins (FLV), seems to contribute to the Kautsky curve [41], as well as other modulators of in vivo Chl-a fluorescence, such as the thylakoidal transmembrane electrochemical potential (Δψ) [33]. Further studies will be necessary to support these hypotheses and characterize the subcellular mechanism(s) involved. Acute exposures to sub-μg/L concentrations of DCMU can lead to other biomolecular effects. Significant modifications in the proteome of *C. reinhardtii* wild type strain CC-125 (mt+) have been observed after 6 h exposure to sub-μg/L doses DCMU (3.3 × 10^−9^ M, about 0.8 μg/L) [42]. The observed proteome variation includes, among others, the upregulation of plastocyanin PCY1 (electron carrier that participates in electron transfer between P700 and the cytochrome b6-f complex in PSI) and the downregulation of LHCA4 (a binding protein part of the light-harvesting complex). Such modification in the *C. reinhardtii* proteome could lead to a functional remodeling of the photosynthetic machinery in exposed cultures.

Overall, future research is required to better investigate mechanisms responsible for the alteration observed in chlorophyll fluorescence parameters following sub-μg/L DCMU exposures, as well as the possible effects on natural communities of green microalgae and/or on freshwater biofilms.

## 5. Conclusions

Chlorophyll *a* fluorescence proved to be a valid short-term in vitro toxicological endpoint to highlight the interfering effects on the *C. reinhardtii* photosynthetic process. Both bioassays presented in this paper are potentially scalable and adaptable for high-throughput applications in ecotoxicity testing of environmental samples. If confirmed, the hypothesized acute ‘stimulatory-like’ effect on the photosynthetic process should carefully be considered for its implication in ecological risk assessment of real scenarios.

## Supplementary Materials

The following are available online at https://www.mdpi.com/article/10.3390/bios12020067/s1, Figure S1: Picture of *C. reinhardii* culture flasks. Details about culture conditions are provided in the main text; Figure S2: Calibration curve of OD_750 nm_ vs. cell count. Each point represents a mean value of five replicate tests (%RSD < 5%); Figure S3: Example of a chlorophyll fluorescence spectra of 2 μg/L DCMU-exposed microalgae cell suspensions (red line) and of blank microalgae cell suspensions (blue line). Details about instrumental setup are provided in the main text.
