# Supplementary material for: Microalgae-Based Fluorimetric Bioassays for Studying Interferences on Photosynthesis Induced by Environmentally Relevant Concentrations of the Herbicide Diuron
Source: Biosensors (Basel). 2022 Jan 25;12(2):67. doi: 10.3390/bios12020067 (PMC8869104; doi:10.3390/bios12020067)
Supplement: Supplementary file 1 [file biosensors-12-00067-s001.zip › biosensors-1551565-supplementary.pdf]

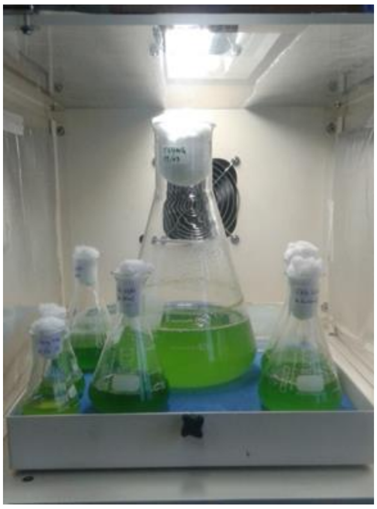

Figure S1. Picture of *C. reinhardii* culture flasks. Details about culture conditions are provided in the main text.

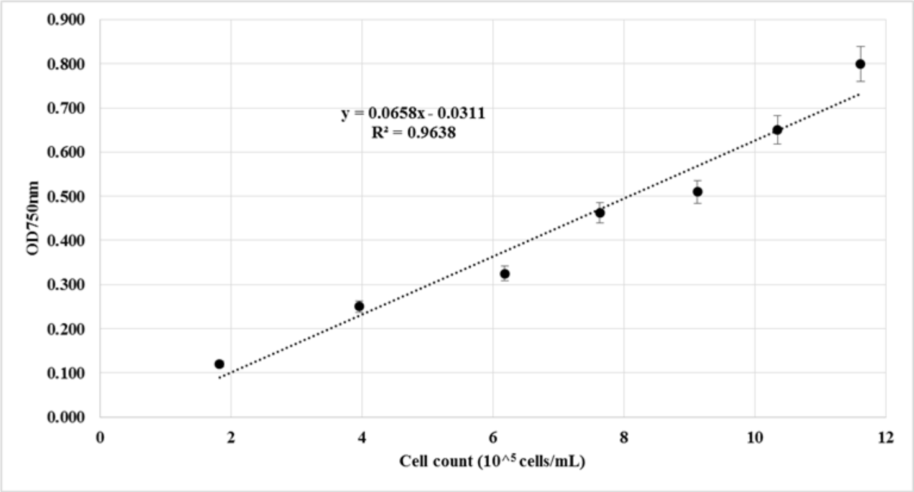

Figure S2. Calibration curve of OD_{750nm} *vs.* cell count. Each point represents a mean value of five replicate tests (%RSD < 5%).

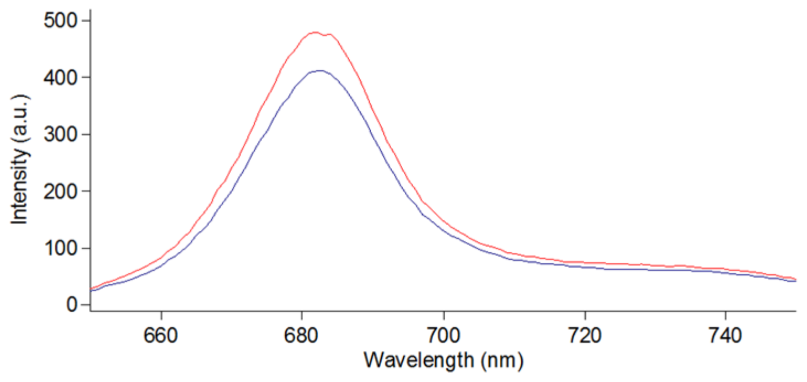

Figure S3. Example of a chlorophyll fluorescence spectra of 2 μg/L DCMU-exposed microalgae cell suspensions (red line) and of blank microalgae cell suspension*s* (blue line). Details about instrumental setup are provided in the main text.
